# Vulvar leiomyoma in an adolescent girl: a case report and review of the literature

**DOI:** 10.1186/s13256-022-03743-7

**Published:** 2023-02-13

**Authors:** Lajya Devi Goyal, Priyanka Garg, Manmeet Kaur

**Affiliations:** 1 Department of Obstetrics and Gynecology, All India Institute of Medical Sciences, Bathinda, 151001 Punjab India; 2 Department of Pathology, All India Institute of Medical Sciences, Bathinda, India

**Keywords:** Vulva, Leiomyoma, Bartholin cyst, Neoplasms, Adolescent

## Abstract

**Background:**

Vulvar leiomyoma is a rare soft tissue tumor, with only around 300 cases described in the literature. Owing to its low incidence of just 0.03% of all gynecological tumors, it often poses a great diagnostic challenge, especially in teenagers. We report this rare occurrence of vulvar leiomyoma in a teenage girl who was primarily left untreated due to cultural taboos and fear of loss of virginity. The main aim in presenting such rare case studies is to raise awareness and expand the diagnostic horizon of the surgeon for appropriate management.

**Case presentation:**

We describe a case of a 15-year-old North Indian, sexually inactive unmarried girl, who presented with a history of painless swelling in the left labia majora for the last 1 year, which gradually increased in size. There was no associated pain or any other difficulty. Local examination revealed a 14 × 10 cm solid, unilateral nonpedunculated mass on the left labia majora with superficial vascularity. Differential diagnoses of sarcoma, lipoma, Bartholin cyst, and fibroid were kept in mind. Ultrasonography revealed a solid mass with superficial vascularity with normal internal genitalia. The mass was enucleated with an intact capsule under anesthesia. Histopathology confirmed it to be benign vulvar leiomyoma. The patient was discharged after 3 days in a satisfactory condition.

**Conclusion:**

Leiomyoma of the vulva is an exceptionally rare tumor and is seldom seen in teenagers. It is often misinterpreted as a Bartholin cyst and should be kept as one of the differential diagnosis in teenage girls presenting with unilateral vulvar swelling. Vulvar leiomyoma can be completely cured by surgical removal if diagnosed timely without compromising virginity, so should never be missed in adolescents.

## Introduction

Leiomyomas are well-circumscribed, benign soft tissue neoplasms of mesenchymal origin, with the uterus being the most frequent site [1]. Extrauterine leiomyomas are extremely rare, and can originate from vulva, vagina, ovaries, urinary bladder, urethra, round ligaments, uterosacral ligaments, inguinal canal, retroperitoneum, sinonasal cavities, and kidney [2]. Leiomyoma of the vulva is extremely rare, accounting for only 0.03% of all gynecological tumors and 0.07% of all vulvar neoplasms [3]. They usually affect females of reproductive age and are seldom seen in adolescents, particularly under 15 years of age [4, 5]. It can be asymptomatic or may have a varied clinical presentation such as painless vulvar swelling associated with erythema or pruritus, and often get misdiagnosed or left untreated. Due to their rare occurrence, vulvar leiomyomas can masquerade as Bartholin cyst or abscess, thus posing a huge diagnostic dilemma. Another diagnostic problem with vulvar masses is differentiation between benign and malignant types, since the majority of them have similar appearance. When encountered with such lesions, detailed history and meticulous examination along with transperineal ultrasound helps to make the diagnosis. Wide local excision of the tumor followed by histopathological examination is the treatment of choice because of the aggressive behavior of other related mesenchymal tumors of the vulva. In low–middle-income countries such as India, lack of awareness among primary healthcare workers and taboos related to female virginity in adolescent girls lead to delayed diagnosis and appropriate management. Although a few hundreds of cases of vulvar leiomyoma have been reported in the literature before, its occurrence in an adolescent girl is a rare finding. We report one such rare case of vulvar leiomyoma in a young unmarried 15-year-old girl who was primarily left untreated by her initial healthcare worker due to lack of knowledge, ignorance, and socio-cultural taboos. To the best of our knowledge, this is one of the first cases reported from India in a teenage girl who was successfully managed without compromising her virginity.

## Case presentation

**Fig. 1:**
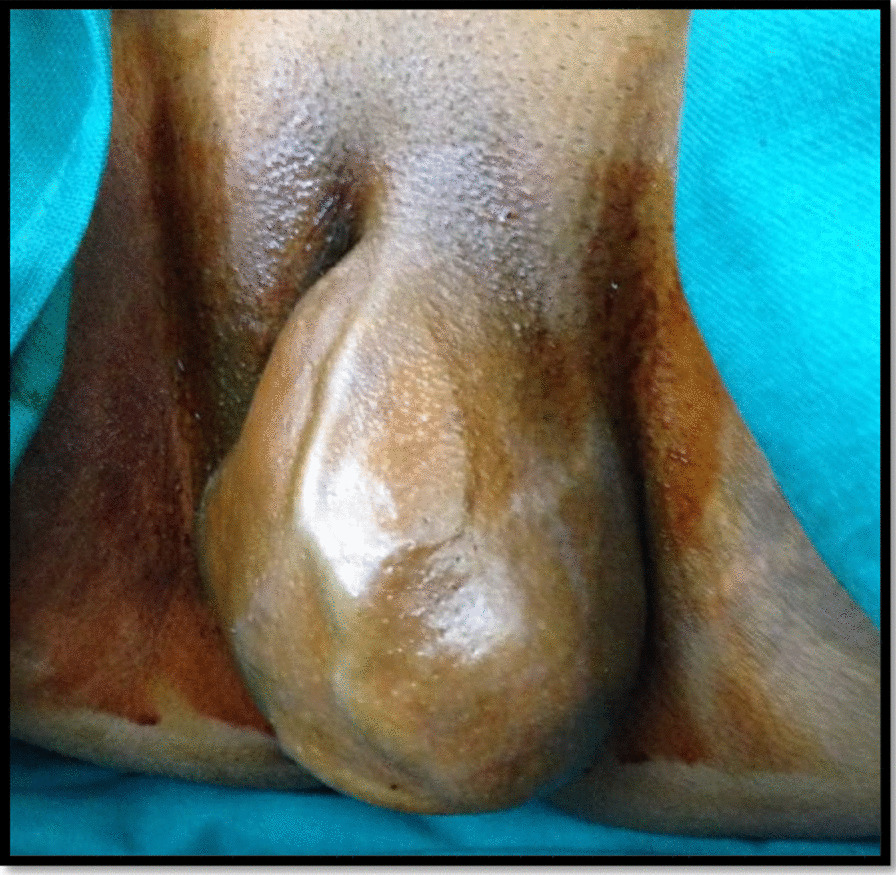
Vulvar swelling on clinical examination

**Fig. 2:**
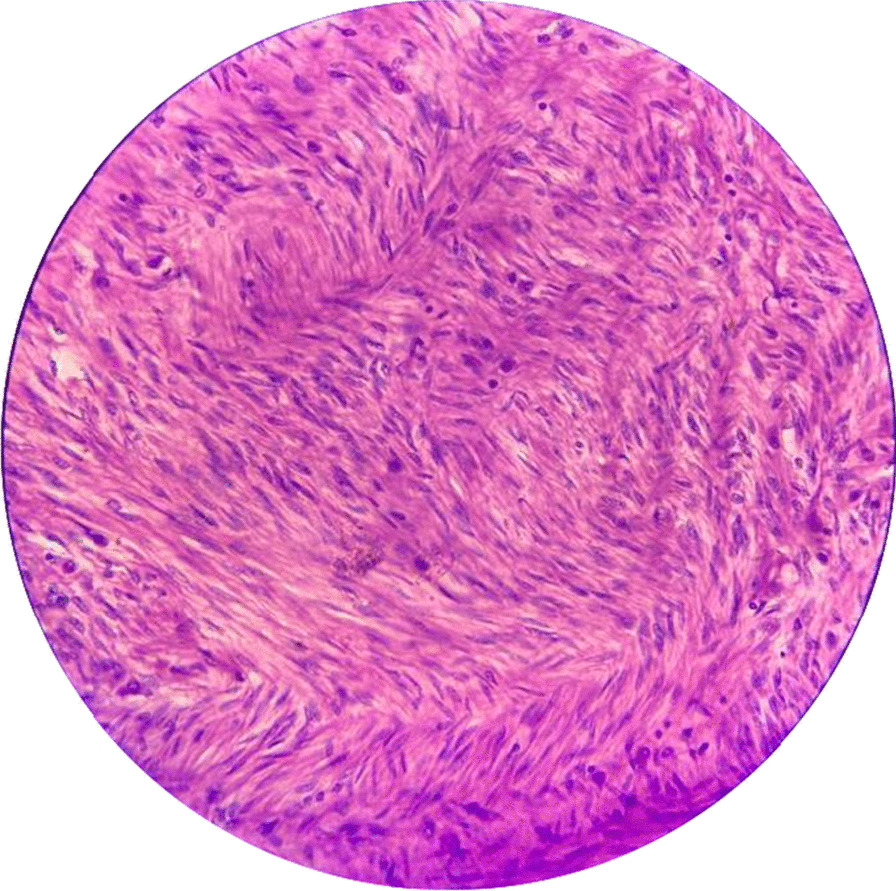
Hematoxylin and Eosin section show an encapsulated tumor comprising mainly oval to spindle-shaped cells exhibiting minimal nuclear atypia and eosinophilic cytoplasm. No area of necrosis or atypical mitosis seen

A 15-year-old North Indian, sexually inactive unmarried girl, presented with a history of painless swelling in the left labia majora for the last year, which gradually increased in size. There was no associated pain or any other difficulty. She attained menarche at the age of 12 years and her menstrual cycles were regular with normal flow. There was no history of vaginal discharge, fever, weight loss, or bowel or bladder complaints. Her past medical and surgical history was unremarkable. The patient went to a primary healthcare worker where she was given some herbal medicine and strictly advised not to undergo surgery as it would affect her virginity. Due to financial constraints and taboos related to loss of virginity, the patient did not seek any further medical advice. On presenting to us, she was in a fair condition with normal systemic examination. Local examination revealed a 14 × 10 cm solid, nonpedunculated mass on the left labia majora with superficial vascularity (Fig. 1). It was slightly warm to touch, nontender, and not adherent to overlying skin or the surrounding structures. Mons and the contralateral labia were apparently normal. There was no associated inguinal lymphadenopathy. The differential diagnoses of sarcoma, lipoma, Bartholin cyst, aggressive angiomyxoma, and fibroid were kept in mind. Transperineal ultrasonography revealed a solid mass with superficial high vascularity with normal internal genitalia. With this clinical presentation and Ultrasonography (USG) findings pointing towards a benign pathology, the decision for surgical excision was taken. Hematological and biochemical investigations were normal. Informed consent was taken for the procedure and publication. Under general anesthesia and aseptic precautions, a longitudinal incision was given on the mucocutaneous junction of the left labia, and the mass was enucleated with an intact capsule. The dead space was obliterated using vicryl no. 1–0 interrupted sutures. The specimen was sent for histopathological examination. Gross examination showed a 12 × 10 cm mass with capsule, 300 g, firm in consistency, grayish–tan in color. On the cut surface, there was a typical whorled appearance without any foci of hemorrhage or necrosis. Microscopy revealed a benign tumor composed of sheets of an oval to spindle cells with no evidence of cellular atypia suggestive of benign vulvar leiomyoma (Fig. 2). Broad-spectrum antibiotics and analgesics were administered in the postoperative period. Her postoperative period was unremarkable. The patient was discharged in a satisfactory condition after 3 days. On follow-up at 6 weeks, and 3 and 6 months, there is no tumor recurrence so far.

## Discussion

Leiomyomas of the female reproductive tract are frequently seen in the uterus, followed by the cervix, round ligament, uterosacral ligament, ovary, and inguinal canal. Their occurrence in the vulva is extremely rare, with < 300 cases reported in the literature since the first case in 1908 [6, 7]. This rare site of occurrence makes our case unique. It originates from smooth muscle cells within erectile tissue, blood vessel walls, round ligament, erector pili muscle, and stem cells located in the Bartholin gland [8]. Another uniqueness of our patient is the age of our patient. The mean age of presentation varies between 30 and 60 years for this tumor, making a case in teenagers even rarer. Our patient was an adolescent, which is a rare age for vulvar leiomyoma to occur. A similar case of vulvar leiomyoma measuring 10 × 10 cm was reported in a 14-year-old girl by Fontinele *et al*. from Brazil [9]. Clinically, the patient presents with a solitary painless swelling, which increases over time, causing difficulty in sitting, walking, or sexual activity. The size of the tumor may range from 0.5 to 15 cm [9]. Our patient presented with a similar finding of painless mass in the vulvar region, which increased in size over 1 year, reaching a size of 14 × 10 cm. Other less common symptoms can be pain due to irritation of peripheral nerves, pruritus, erythema, and occasional compression of the bladder or rectum [3]. Roy *et al*. reported a case of vulvar leiomyoma who presented with severe itching over the vulvar swelling [3]. The simultaneous development of vulvar leiomyomas and leiomyomas at other sites is not uncommon. Wahlen *et al*. reported an interesting case of vulvar leiomyoma coexisting with leiomyoma in the esophagus in a mother and her daughter from Sweden [6]. The usual site to be involved is labia majora and clitoris, with occasional involvement of labia minora. Vulvar leiomyomas are usually firm to feel but sometimes the consistency may be soft cystic due to degenerative changes. Thus, initial diagnosis is often challenging due to infrequent occurrence and nonspecific clinical presentation. The differential diagnosis includes other solid or cystic masses in the vulva such as Bartholin cyst or abscess, lipoma, fibroma, schwannoma, aggressive angiomyxoma, hemangioma, vulvar cyst, leiomyosarcoma, and dermatofibrosarcoma [9–11]. The most common confusion occurs with Bartholin cyst due to topography of the Bartholin gland [9]. The clinical features such as everted labia minora and cystic swelling favors the diagnosis of Bartholin cyst or abscess, while inverted minora and firm consistency of the swelling point towards other vulvar tumor. When we examined our patient, a firm mass was located on the left labia majora, which was nonfluctuating, nontender, mobile, and regular in shape, suggestive of a benign solid lesion. Diagnosis can be made by ultrasonography (transperineal), which is the initial investigation of choice to evaluate vulvar leiomyomas because of its low cost, high accessibility, and sensitivity in establishing the accurate diagnosis. The next challenge is to differentiate between benign and malignant lesions. Magnetic resonance imaging (MRI) is used sparingly to delineate the extent of the tumor and differentiate benign from malignant lesions. A malignant growth shows low-intensity signal on T2-weighted scans unlike normal smooth muscle cells [12]. However, histopathology of the surgical specimen is the gold standard for definitive diagnosis and to exclude any malignancy. Smooth muscle tumors of the vulva are categorized into three types: leiomyomas, atypical leiomyomas, and leiomyosarcomas. Tavassoli and Norris proposed criteria to differentiate leiomyosarcoma from atypical and benign tumors. These characteristics include: tumor size more than 5 cm in the largest dimension, infiltrative margins, and more than five mitotic figures per ten high-power fields [13]. Nielsen *et al*. added a fourth criterion of moderate-to-severe cytological atypia. They suggested that if three or all features are present, the neoplasm is a sarcoma, whereas atypical benign leiomyomas satisfy only two characteristics, and benign leiomyomas exhibit only one or none of these traits [14]. Our case did not exhibit any of these attributes, which indicated the diagnosis of a benign tumor.

The definitive treatment is always enucleation and wide local excision of the tumor with adjacent normal tissue to avoid recurrences in the future. Selective receptor modulators may be beneficial as an adjuvant to surgery as the tumor stains positively for estrogen and progesterone receptors on immunohistochemistry [12]. Nielson *et al*. studied 25 cases of smooth muscle tumors of the vulva presenting with painless mass. Most of the cases masqueraded as Bartholin gland cyst. However, on histopathology, 20 were diagnosed to be leiomyoma and five leiomyosarcomas. On follow-up of these patients, only one patient with vulvar leiomyoma had recurrence after 10 years. Kothandaraman *et al*. reported a case of vulvar leiomyoma with recurrence after 4 years of primary excision [2]. Hence, the chances for recurrence and the need for regular long-term follow-up need to be explained to the patient [14]. Thus, even with benign tumors, continued surveillance for recurrence should be done.

## Conclusions

Vulvar leiomyomas are a rare entity particularly in teenagers, posing a diagnostic dilemma and often being mistaken for Bartholin gland cyst. A thorough medical workup along with imaging studies is required for the appropriate diagnosis. Treatment of choice is surgical removal without compromising virginity in teenage girls, and final diagnosis is made after histopathological examination only. The main aim in presenting such rare case studies is to expand the diagnostic horizon of the surgeon for appropriate treatment.

## Data Availability

Not applicable.
